# Characterization and validation of potential therapeutic targets based on the molecular signature of patient-derived xenografts in gastric cancer

**DOI:** 10.1186/s13045-018-0563-y

**Published:** 2018-02-13

**Authors:** Zuhua Chen, Wenwen Huang, Tiantian Tian, Wanchun Zang, Jingyuan Wang, Zhentao Liu, Zhongwu Li, Yumei Lai, Zhi Jiang, Jing Gao, Lin Shen

**Affiliations:** 1 Key Laboratory of Carcinogenesis and Translational Research (Ministry of Education/Beijing), Department of Gastrointestinal Oncology, Peking University Cancer Hospital and Institute, Fu-Cheng Road 52, Hai-Dian District, Beijing, 100142 China; 2 Novogene Bioinformatics Institute, Beijing, China; 3 Key Laboratory of Carcinogenesis and Translational Research (Ministry of Education/Beijing), Department of Pathology, Peking University Cancer Hospital and Institute, Beijing, China

**Keywords:** PDX model, Advanced gastric cancer, Molecular signature, Therapeutic targets

## Abstract

**Background:**

Patient-derived xenograft (PDX) models with definite molecular signature are attractive preclinical models for development of novel targeted drugs. Here, we profiled and explored potential therapeutic targets based on characterized PDX models for advanced gastric cancer (AGC).

**Methods:**

The genomic variation and molecular profile of 50 PDX models from AGC patients were analyzed by targeted next-generation sequencing, in situ hybridization, and immunohistochemistry. The antitumor activities of several targeted drugs were investigated in the PDX models. Furthermore, response biomarkers were explored.

**Results:**

Each PDX model had individual histopathological and molecular features, and recurrent alterations in the MAPK, ErbB, VEGF, mTOR, and cell cycle signaling pathways were major events in these PDX models. Several potential drug targets, such as EGFR, MET, and CCNE1, were selected and validated in this study. Volitinib demonstrated strong antitumor activity in PDX models with MET and phosphorylated MET (pMET) overexpression. The EGFR monoclonal antibodies BK011 and cetuximab inhibited tumor growth in a PDX model with *EGFR* amplification. Afatinib inhibited tumor growth in the PDX models with *EGFR* amplification, EGFR overexpression, or *HER2* amplification. Apatinib was more sensitive in the PDX models with high microvessel density. The CDK1/2/9 inhibitor AZD5438 had superior anti-tumor activity in two models with higher copy number of *CCNE1*.

**Conclusions:**

PDX models with defined molecular signature are useful for preclinical studies with targeted drugs, and the results should be validated in larger studies with PDX models or in clinical trials.

**Electronic supplementary material:**

The online version of this article (10.1186/s13045-018-0563-y) contains supplementary material, which is available to authorized users.

## Background

In China, more than 70% of patients with gastric cancer (GC) are diagnosed at an advanced stage, and the patients have a very poor overall survival due to rather few available therapeutic drugs and frequent drug resistance [1]. Fluorouracil-based combination chemotherapy has been the main treatment for advanced gastric cancer (AGC) for a long time, with unsatisfactory clinical responses, and many with novel drugs failed in clinical trials in GC [2–4]. Therefore, it is urgent to explore new therapeutic targets and to develop new GC drugs.

Appropriate animal models are very important for evaluation of novel drug candidates. Patient-derived xenograft (PDX) models are attractive models for preclinical studies due to higher comparability of their biological characteristics with the primary tumors of the patients [5, 6]. Based on our previous study, PDX models using real-time gastroscopic biopsies in GC were successfully established and characterized for the first time, providing an attractive platform for preclinical studies of novel and approved drug candidates in AGC [7].

Our previous research mainly focused on the establishment, characterization of pathological features, and chemosensitivity of the PDX models [7]. Currently, individualized treatment guided by genotyping or expression profiling is the major model of precision medicine, and in order to facilitate the quick and precise use of PDX models in preclinical studies of new drugs, the genomic profiles and expression profiles of some critical molecules of PDX models will be analyzed in this study. Moreover, some potential therapeutic targets will be validated using the corresponding inhibitors.

## Methods

### PDX sample collection and genomic DNA extraction

Frozen tumor tissues were obtained from 50 PDX models, and genomic DNA was extracted using a QIAamp DNA Mini Kit (Qiagen, Hilden, Germany) according to the manufacturer’s instructions. The quantification of genomic DNA samples were assessed with a Nanodrop 2000 Spectrophotometer (Thermo Fisher Scientific Inc., Wilmington, DE, USA).

### Target enrichment of genomic DNA and sequencing

Genomic DNA was fragmented into segments of 150–300 bp by a Covaris S220 instrument (Covaris, Inc., Woburn, MA, USA). The DNA libraries were created using a KAPA Hyper Prep Kit (Kapa Biosystems, Boston, MA, USA), followed by Agilent’s SureSelectXT Target Enrichment System for Illumina Paired-End Sequencing Library Protocol (Agilent Technologies, Santa Clara, CA, USA). The DNA libraries were quantified by an Agilent QPCR NGS Library Quantification Kit (Agilent Technologies), and DNA libraries with average insert sizes of 150 bp were sequenced on an Illumina HiSeq 2000 instrument (Illumina, San Diego, CA, USA).

### Sequencing quality control and alignment

Quality control (QC) was conducted by filtering out the adapter sequences and low-quality reads, and the ultimate Q20 and Q30 of the samples were 95.6 and 90.5%, respectively (Additional file 1: Table S1). The sequencing reads were aligned with the reference human genome (build hg19) using the Burrows-Wheeler Alignment (BWA) program with default parameters. PCR duplications were marked with Picard tools (http://broadinstitute.github.io/picard/). According to the results of the alignment, the coverage of the target region was more than 99%, and the mapping rate was typically no less than 95%.

### Gene variant calling

The workflow of gene variation calling is shown in Additional file 2: Figure S1. Single-nucleotide variants (SNVs), insertions or deletions (InDels), and fusion genes were called using Samtools (http://www.htslib.org/) and a customized software-NovoFusion. The mutations were annotated by the ANNOVAR (http://annovar.openbioinformatics.org/en/latest/) with information from the COSMIC (http://cancer.sanger.ac.uk/cosmic) and ClinVar (https://www.ncbi.nlm.nih.gov/clinvar/) databases. In our study, copy number variations (CNVs) analysis was performed with the Event-wise testing algorithm based on read depth of coverage according to a previous report [8]. Several lymphocyte samples were used as reference sets to provide a neutral copy number level.

### GO enrichment and pathway analysis

The Gene Ontology (GO) enrichment and Kyoto Encyclopedia of Genes and Genomes (KEGG) analysis of the genes identified in more than two PDX models were conducted using the Database for Annotation, Visualization and Integrated Discovery Bioinformatics Resources 6.7 (DAVID; http://david.abcc.ncifcrf.gov). Potential inhibitors targeting these pathways were selected. These compounds are either undergoing investigation in preclinical studies or in clinical trials.

### Genomic landscape of GC across PDX models and TCGA data

We downloaded somatic SNVs and CNAs from The Cancer Genome Atlas (TCGA) research network (http://cancergenome.nih.gov/). A genomic landscape analysis of GC across the PDX models and TCGA data was conducted in cBioportal [9, 10] (http://www.cbioportal.org/).

### Immunohistochemistry and fluorescent in situ hybridization

Formalin-fixed and paraffin-embedded tissue blocks of the 50 PDX models were prepared. Candidate targets, including EGFR, HER3, MET, and PD-L1, were stained via immunohistochemistry (IHC) using anti-EGFR antibody (#4267, Cell Signaling Technology, Danvers, MA, USA), anti-HER3 antibody (#2708, Cell Signaling Technology), anti-MET antibody (#790-4430, Ventana Medical Systems, Tucson, AZ, USA), and anti-PD-L1 antibody (#M4420, Spring Bioscience Corp., Pleasanton, CA, USA) according to the manufacturers’ instructions. IHC results were evaluated according to a previously published method [11–13]. Fluorescent in situ hybridization (FISH) was performed for *MET* and *EGFR* genes using the MET/CEN7 Dual Color Probe Kit (Zytovision, Bremerhafen, Germany) and EGFR/CEN7 Dual Color Probe (Zytovision) according to the manufacturer’s instructions. Gene amplifications were defined as the ratio of *MET*/CEN7 ≥ 2.2 and *EGFR*/CEN7 ≥ 2.2. All of the IHC and FISH results were reviewed and scored by two independent pathologists blinded to each other.

### Epstein-Barr virus-encoded RNA in situ hybridization and microsatellite instable detection

EBV-encoded RNA (EBER) in situ hybridization (ISH) was performed using INFORM EBER Probe (Ventana, Tucson, AZ, USA) [14]. A tumor was considered to be EBER-positive if the signal was observed in 20% or more of the tumor cells. The results of EBER ISH were assessed by two independent specialists blinded to each other.

The microsatellite instable (MSI) status of the PDX models was evaluated by the expression of mismatch repair (MMR) proteins by immunohistochemical analysis. Monoclonal antibodies specific for MLH1, PMS2, MSH2, and MSH6 were obtained from GeneTech, Inc., Shanghai, China. As positive controls stromal cells were used. The loss of MMR protein expression was defined as the absence of nuclear staining in neoplastic epithelial cells. The IHC results were assessed by two independent specialists blinded to each other.

### Real-time PCR

The detection of *CCNE1* amplification by quantitative real-time PCR was performed with a CCNE1 TaqMan Copy Number Variation Assay (Applied Biosystems of ThermoFisher, Waltham, MA) and a RNase P TaqMan Copy Number Reference Assay (Applied Biosystems). Human genomic DNA was used as a control. The ratio of *CCNE1*/*TERT* was calculated by the CopyCaller™ Software v 1.0 (Applied Biosystems) using the comparative Ct (ΔΔCt) method.

### Evaluation of the drug response of targeted drugs in the PDX models

Target candidates were selected based on the molecular signatures of the 50 PDX models, and in vivo experiments were performed to evaluate the antitumor activity of several novel inhibitors, including (1) volitinib (kindly provided by AstraZeneca Pharmaceuticals, Cambridge, UK) targeting MET; (2) BK011 (kindly provided by Newind Biotech, Inc., Zhejiang, China) (The synthesis details and product characterization of BK011 are shown in Additional file 3: methods, Additional file 4: Figure S2 and Additional file 5: Table S2), cetuximab (purchased from Merck KGaA, Darmstadt, Germany) and afatinib (kindly provided by Boehringer Ingelheim GmbH, Ingelheim am Rhein, Germany) targeting EGFR; (3) apatinib (kindly provided by Jiangsu Hengrui Medicine Co., Ltd., Jiangsu, China) targeting vascular endothelial growth factor receptor-2 (VEGFR2); (4) AZD5438 (purchased from Selleck Chemicals, Houston, TX, USA) targeting CDK1/2/9; (5) Paclitaxel (purchased from Peking Union Pharmaceuticals, Beijing, China). All procedures were performed under sterile conditions at an SPF facility.

Tumors were subcutaneously implanted into NOD/SCID mice, and when the tumor volume reached a volume of 150–250 mm^3^, mice were randomly assigned to different groups (*N* = 5 mice/group): (1) the control group, physiological saline 100 μl treatment by daily oral gavage/ intraperitoneal injection; (2) the volitinib group, 30 mg/kg daily by oral gavage; (3) the BK011 group, 50 mg/kg twice a week by intraperitoneal injection; (4) the cetuximab group, 50 mg/kg twice a week by intraperitoneal injection; (5) the afatinib group, 15 mg/kg daily by oral gavage; (6) the apatinib group, 150 mg/kg daily by oral gavage; (7) the AZD5438 group, 20 mg/kg daily by oral gavage; (8) the paclitaxel group, 5 mg/kg twice a week by intraperitoneal injection. All of the animals were treated for three weeks, and the tumor sizes and body weights of the mice were measured every two days. The tumor volume (V) and tumor growth inhibition (TGI) were calculated using the following formulas: V = L × W^2^/2 (L, length and W, width), and TGI = [1-(ΔT/ΔC)] × 100% (ΔT = mean tumor volume changes in the drug treatment group and ΔC = mean tumor volume changes in the control group). According to previous reports [15, 16], PDX models were classified as high-responder with TGIs > 60% and poor-responders with TGIs < 30%.

After the mice had been sacrificed, we conducted IHC and immunoblot to assess the expression of various markers using anti-pEGFR antibody (#3777), anti-HER2 antibody (#2165),anti-pHER2 antibody (#2243), anti-AKT antibody (#4691), anti-pAKT antibody (#4060), anti-ERK antibody (#4695), anti-pERK antibody (#4370), anti-S6 antibody (#2217), anti-pS6 antibody (#4858), and anti-CD31 antibody (#77699) purchased from Cell Signaling Technology. Anti-β-actin antibody (Lot #014 M4759) was purchased from Sigma-Aldrich (St. Louis, MO, USA).

### Statistical analysis

Statistical analysis was performed using SPSS v 21.0 software (IBM SPSS Inc., Armonk, NY, USA). The differences in alterations of clinical pathological features were analyzed using the Mann-Whitney test and the Kruskal-Wallis test. The differences of alteration rates in different subgroups were evaluated by the chi-square test. For the in vivo study, the differences between the groups were analyzed using the unpaired 2-tailed *t* test or 1-way ANOVA. *P* < 0.05 was considered to be statistically significant.

## Results

### Identification of variants and pathway enrichment in 50 PDX models

**Fig. 1:**
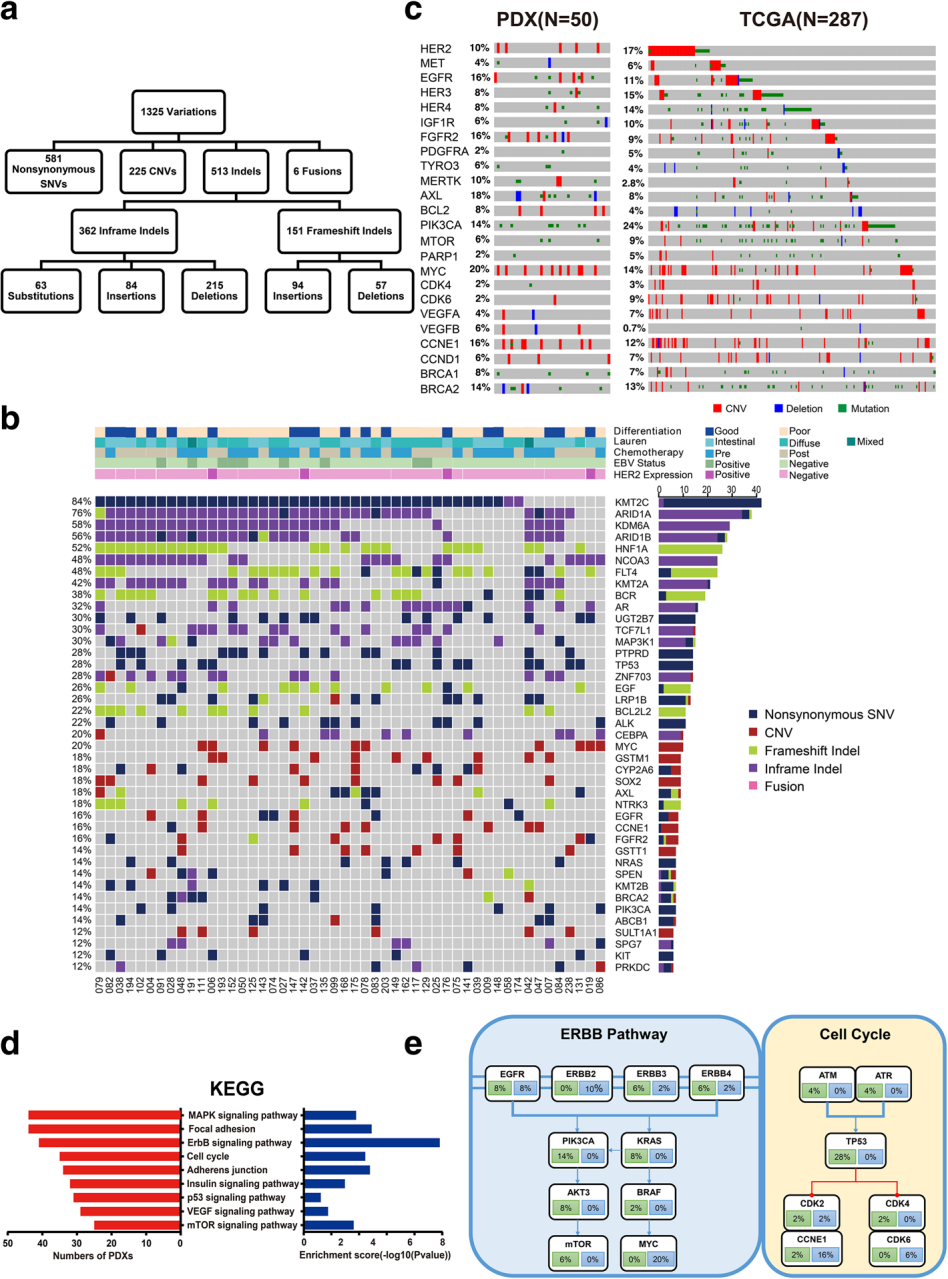
Identification of variants and pathway enrichment in PDX models. **a** We detected 1325 variations, including 581 nonsynonymous single-nucleotide variations, 225 CNVs, 513 indels, and 6 translocation fusions. **b** Genomic alterations were analyzed with different clinicopathological features. Navy, non-synonymous SNV; dark red, CNVs; yellow-green, frameshift indel; purple; inframeshift indel; and pink, fusion. **c** Genomic landscape analysis of genes altered in the PDX and TCGA datasets. Red, amplification; blue, deletion; and green, mutation. **d** Several relevant pathways, including the MAPK, ErbB, cell cycle, mTOR, and VEGF, were found to be enriched. **e** Details for molecular alterations involved in the ErbB and cell cycle pathway. Blue, amplification; green, mutation

A panel of 483 genes (Additional file 6: Table S3) was sequenced for the 50 PDX models. Consistent with other reports on PDX models derived from hepatocellular carcinoma [17], a significant proportion of sequence reads originated from mice. The average coverage depth in this study was 541-fold, a total of 1325 variations in the PDX models from human sequence reads were called, including 581 non-synonymous SNVs, 225 CNVs, 513 indels, and 6 translocation fusions (Fig. 1a). Clinicopathological features and detailed alterations for each PDX model are summarized in Additional file 7: Table S4 and Additional file 8: Table S5, and these results suggest that each PDX had unique genomic make-up.

According to the genomic alterations of the 50 PDX models (Fig. 1b), the top altered genes included *KMT2C*, *ARID1A*, *KDM6A*, *HNF1A*, and *NCOA3*, which, except for *ARID1A*, have so far rarely been reported in GC, and deletions dominated, except for *KMT2C*. Furthermore, a substantial amount of well-known alterations, including *TP53* (28%), *KRAS* (8%), *HER2* (10%), *EGFR* (16%), *HER3* (8%), *ERBB4* (8%), *MET* (4%), *IGF1R* (6%), *CCNE1* (16%), *MYC* (20%), *BRCA1* (8%), *BRCA2* (14%), and *PIK3CA* (14%), were identified, and they were remarkably consistent with the TCGA data (Fig. 1c). Many of these well-known alterations might be potential targets, which will be analyzed in following studies.

In this study, a total of 207 genes were identified as having mutations in more than two PDX models. Next, GO and KEGG pathway analyses using DAVID Bioinformatics Resources 6.7 were performed. Based on the results of the GO analysis, these alterations were significantly associated with protein phosphorylation (ontology: biological process), membrane fraction (ontology: cellular component), and protein tyrosine kinase activity (ontology: molecular function). Several important pathways, including MAPK, ErbB, cell cycle, mTOR, and VEGF were enriched based on our results (Fig. 1d), which suggested potential directions for drug development. For instance, ErbB and cell cycle signaling pathways were altered in 82% and 70% of the 50 PDX models, respectively, and the detailed molecular alterations involved in the corresponding pathways are shown in Fig. 1e. Already several specific inhibitors targeting these pathways have been developed or are in various stages of development [18–20].

### Expressions of several critical molecules in GC

**Fig. 2:**
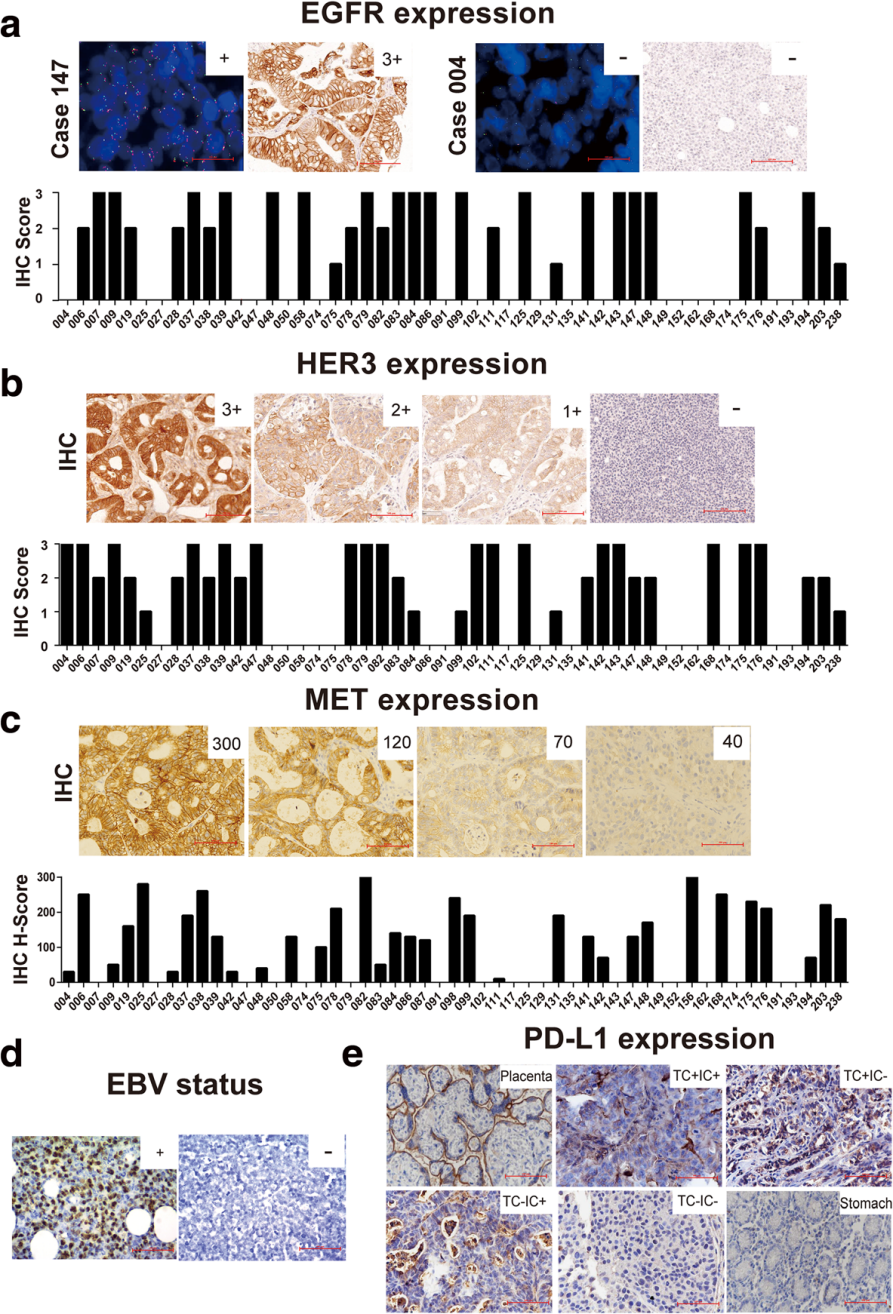
Expressions of several critical molecules in GC PDX models. **a** EGFR expression was evaluated with scores of: 0, 1+, 2+, and 3+. For the FISH assay, the red and green signals represented *EGFR* and CEN7, respectively. Scale bar represents 100 μm. **b** HER3 expression was evaluated with scores of: 0, 1+, 2+, and 3+. Scale bar represents 100 μm. **c** MET expression was evaluated with H-scores. Scale bar represents 100 μm. **d** EBV infection status was detected by EBV-encoded RNA in situ hybridization. EBER-positive cells were observed in 20% or more of the tumor cells. Scale bar represents 100 μm. **e** PD-L1 expression was detected in the placenta (positive control), normal gastric tissue (negative control), and PDX models. Patterns with ≥ 5% positive tumor cells or immune cells was considered to be PDL-1 positive. Scale bar represents 100 μm

Up to now, the only clear therapeutic target for GC is HER2. However, there are still a few critical molecules, including EGFR, HER3, MET, and PD-L1, which have been used as potential targets and were verified in our study. Our data demonstrated that *EGFR* amplification occurred in two of the 50 PDX models (4%) as demonstrated by FISH. EGFR and HER3 expression were found in 30 (60%) and 33 (66%) of the 50 PDX models, respectively (Fig. 2a, b). *MET* amplification was not observed in our study, and c-MET expression was detected in 31 (62%) of the 50 PDX models (Fig. 2c). PDX models with specific molecular features will be useful in future preclinical studies.

Immunotherapy targeting PD-1/PD-L1 is widely pursued preclinically and clinically. Several indicators, including the EBV infectious status, PD-L1 expression, and MSI status, are considered to be associated with response, and were therefore analyzed in this study. In total, nine PDX models were shown to be infected with EBV (Fig. 2d) and designated as EBV-positive GC (18%), which was higher than that (9%) in TCGA. Based on the TCGA data, EBV-positive GC cases feature *PD-L1* or *PD-L2* amplification. PD-L1 expression, rather than *PD-L1* amplification, was detected in 16 of the 50 PDX models (32%), of which 18% appeared in both tumor cells and immune cells, 8% appeared only in tumor cells, and 6% appeared only in immune cells (Fig. 2e). Consistent with the TCGA data and other reports [21], PD-L1 expression in the EBV-positive PDX models was higher than in the EBV-negative models (7/9 vs 9/41, *P* = 0.004 by chi-square test) (Additional file 9: Table S6).

### Validation of potential therapeutic targets in specific PDX models

**Fig. 3:**
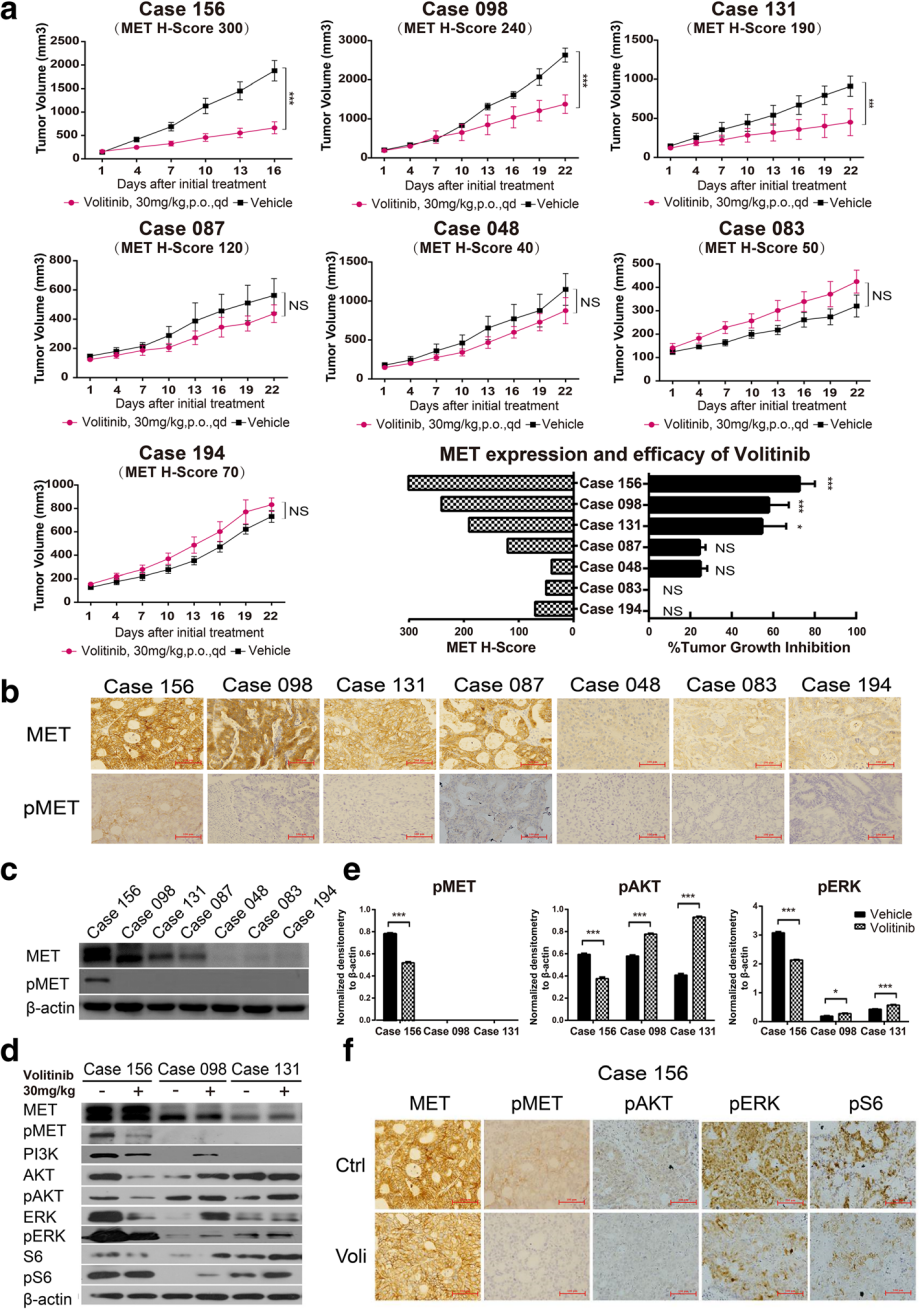
Efficacy of volitinib on PDX models with the corresponding expression of MET and pMET. **a** Volitinib showed significant antitumor activity in three out of seven PDX models (*n* = 5 per group). Tumor volumes and proportion of tumor growth inhibition were expressed as means ± SD. NS, *p* > 0.05; ***p < 0.001 according to repeated measures ANOVA. **b**, **c** The MET and pMET expression of corresponding PDX models assessed by IHC and immunoblot. Scale bar represents 100 μm. **d** The immunoblot analysis of critical molecules in the PI3K/AKT pathway before and after treatment with volitinib. **e** Quantification and normalization of immunoblot bands of pMET, pAKT, and pERK. *p < 0.05, ***p < 0.001 according to unpaired two-tailed *t* test. **f** Immunohistochemical analysis showed that volitinib reduced the level of phosphorylated MET, AKT, ERK, and S6 in case 156. Scale bar represents 100 μm

According to the above results, several potential targets and pathways were identified from the PDX models, which were further analyzed. Volitinib, a tyrosine kinase inhibitor against MET, showed selective antitumor activity in PDX models with high MET expression, especially in the PDX models with MET/pMET overexpression (Fig. 3a–c). From seven PDX models, only case 156 was a high-responder with pMET expression, which suggested that pMET expression might be a predictive biomarker for volitinib. Moreover, after the treatment with volitinib, MET downstream signaling pathways were inhibited as indicated by the reduction of phosphorylated AKT, ERK, and S6 in case 156 (Fig. 3d–f).

**Fig. 4:**
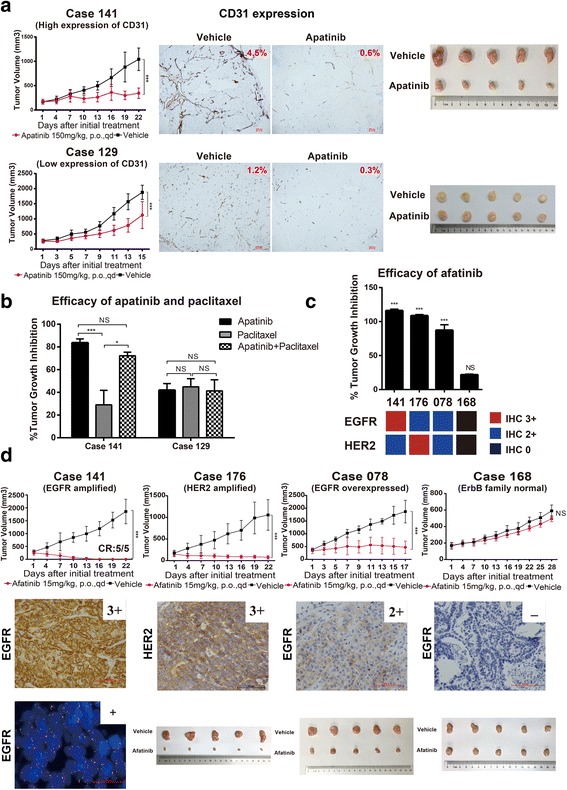
Efficacy of apatinib and afatinib in PDX models. **a** Therapeutic response of apatinib with expressions of CD31 and corresponding tumor volumes before and after treatment (*n* = 5 per group). Tumor volumes were expressed as means ± SD. ***p < 0.001 according to repeated measures ANOVA. Scale bar represents 100 μm. The expression of CD31 was scored as the percentage of positive tumor cells divided by the total number of tumor cells examined, which were analyzed by the Aperio ImageScope software v8.2.5. **b** The efficacy of apatinib monotherapy and combination with paclitaxel in two PDX models. NS, *p* > 0.05; *p < 0.05, ***p < 0.001 according to repeated measures ANOVA. **c** The efficacy of afatinib in four PDX models with different expression levels of EGFR and HER2. The proportion of tumor growth inhibition were expressed as means ± SD. NS, *p* > 0.05; ***p < 0.001 according to repeated measures ANOVA. **d** Afatinib induced tumor growth inhibition in *EGFR*-amplified (case 141), EGFR-overexpressed (case 078), and *HER2*-amplified (case 176) PDX models (*n* = 5 per group). Tumor volumes were expressed as means ± SD. NS, *p* > 0.05, ***p < 0.001 according to repeated measures ANOVA. CR, complete regression. Scale bar represents 100 μm

Apatinib, a tyrosine kinase inhibitor targeting VEGFR2, has been approved as third-line treatment for patients with AGC [20]. However, the patient segment that may benefit most from apatinib is not known. In this study, we randomly selected two PDX models to evaluate the efficacy of apatinib and the microvessel density before and after apatinib treatment. However, PDX models with high microvessel density (analyzed by CD31 expression) were relatively more sensitive to apatinib, and the microvascular density was reduced after apatinib treatment (Fig. 4a), which suggested that microvessel density indicated by CD31 may be used as a predictor for apatinib treatment. Moreover, we did not observe synergistic effects between apatinib and paclitaxel (Fig. 4b).

Afatinib is a pan-HER inhibitor that has been approved for platinum-refractory advanced lung squamous cell cancer [22]. The antitumor activity of afatinib in patients with AGC was not known. We found that afatinib could inhibit tumor growth in PDX models with EGFR amplification (case 141), EGFR overexpression (case 078), or HER2 amplification (case 176) (Fig. 4c, d). Afatinib did not inhibit tumor growth of PDX models without alterations of the EGFR family members (case 168). The underlying mechanisms are still being investigated.

**Fig. 5:**
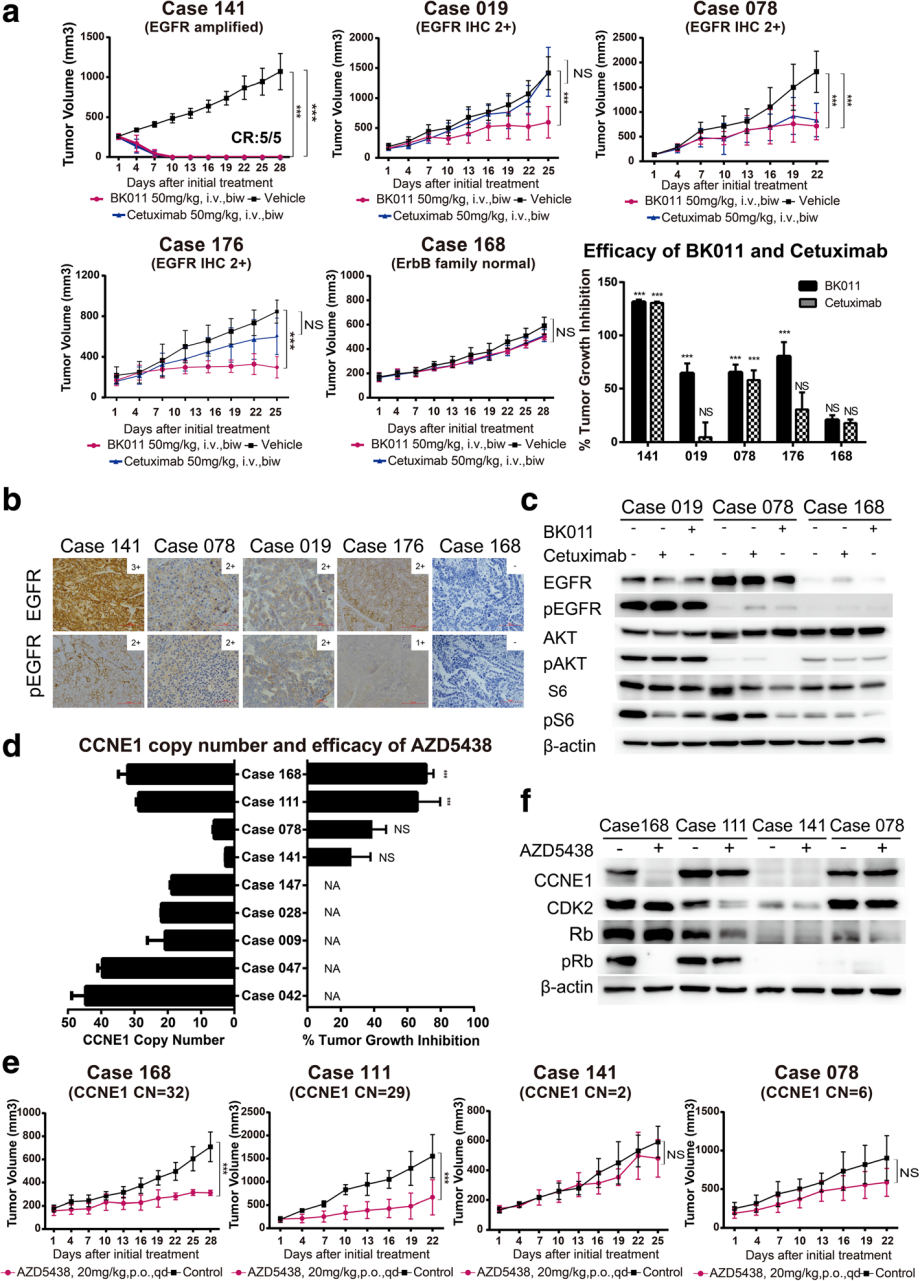
*EGFR* and *CCNE1* amplifications could be potential biomarkers of therapy targeting EGFR and CDK2. **a** The efficacy of BK011 and cetuximab on five PDX models (*n* = 5 per group). Tumor volumes and proportion of tumor growth inhibition were expressed as means ± SD. NS, *p* > 0.05; ***p < 0.001 according to repeated measures ANOVA. CR, complete regression. **b** The immunohistochemical analysis of EGFR and phosphorylated EGFR expression in five PDX models. Scale bar represents 100 μm. **c** The expression and phosphorylation of EGFR, AKT, and S6 after BK011 or cetuximab treatment assessed by immunoblot. **d** *CCNE1* copy numbers of eight PDX models quantified by real-time PCR and the tumor growth inhibition of AZD5438 in four PDX models. The copy number and proportion of tumor growth inhibition were expressed as means ± SD. NS, *p* > 0.05; ***p < 0.001 according to repeated measures ANOVA. **e** AZD5438 showed potent antiproliferative activity in two PDX models with *CCNE1* copy numbers ≥ 29 alterations (*n* = 5 per group). Tumor volumes were expressed as means ± SD. NS, *p* > 0.05; ***p < 0.001 according to repeated measures ANOVA. **f** AZD5438 exerted antitumor effect in case 168 accompanied by reduction of expression of CDK2, CCNE1, and phosphorylated retinoblastoma (pRb). Whereas in case number 111, slight reduction of CDK2 but no impact on level of CCNE1 or pRB

Cetuximab, a monoclonal antibody against EGFR, was confirmed to have limited antitumor activity by the EXPAND trial in GC without stratification [23]; however, it showed efficacy in some specific groups, such as for patients with *EGFR* amplification and overexpression [24]. BK011 is another monoclonal antibody against EGFR, and its therapeutic effect, as well as that of cetuximab, were explored in this study. Both BK011 and cetuximab exerted potent antitumor activity in PDX models with *EGFR* amplification and moderate or mild antitumor activity in PDX models with EGFR overexpression (Fig. 5a, b). BK011 and cetuximab exerted antitumor activities by inhibiting the phosphorylation of AKT and S6 rather than EGFR (Fig. 5c).

Cyclin-dependent kinase 2 (CDK2) was reported as an ideal target for high-grade serous ovarian cancer (HGSC) with elevated CCNE1 expression [25]. In our study, *CCNE1* was amplified in eight PDX models validated by real-time PCR (CNV 6 to 48) (Fig. 5d). AZD5438, a CDK1/2/9 inhibitor, has shown potent antiproliferative activity in a range of tumor cells [26]. We evaluated the efficacy of AZD5438 in four PDX models with or without *CCNE1* amplification (Fig. 5d, e), and we found that AZD5438 exerted an antitumor effect by inhibiting the expression of CDK2, CCNE1, and phosphorylated retinoblastoma (pRb) in PDX models with a high *CCNE1* copy number (Fig. 5f). However, in PDX models with low *CCNE1* copy number, neither Rb expression nor changes in CDK2/CCNE1 expression were observed. AZD5438 had superior anti-tumor activity in models with higher copy number of CCNE1.

## Discussion

The discovery of new targeted drugs for the treatment of cancer is growing vigorously. In order to improve the drug discovery success rate, each step involved in the drug discovery process should be strictly controlled. A preclinical study is critical before new drugs explored in clinical trials, which means that predictive preclinical animal models are of great relevance. PDX models have become a favored model in preclinical studies due to their superiority compared with traditional cell line-based models and the high consistency of biological characteristics with the primary tumors of the patients [5, 6].

We established large number of PDX models using real-time gastroscopic biopsies in GC with different characteristics [7], and some PDX models have already been used in research [27, 28]. In this study, the molecular profile of alterations and expressions of our PDX models were analyzed in detail to find potential therapeutic targets. Several important pathways, including the MAPK, ErbB, cell cycle, mTOR, and VEGF, were enriched in our study, and several alterations were identified, which were consistent with a corresponding TCGA dataset from primary GC cases. Based on literature suggestions, several potential targets were selected and validated in our study.

Receptor tyrosine kinases (RTKs) have been reported to be key regulators of cellular processes in the development and progression of various cancers [29]. Dysregulation of the MET signaling pathway has been reported to occur in a variety of cancers, with correlations to poor clinical outcomes and drug resistance. Compared with the gene amplification of *MET*, the incidence of protein overexpression was more common in GC [11, 30, 31], but the correlation of MET expression with the therapeutic response to volitinib was not known. In our study, seven PDX models were used to evaluate the efficacy of volitinib, and volitinib demonstrated strong antitumor activity in the PDX models with MET and pMET overexpression by inhibiting the PI3K/mTOR pathway, which suggested that patients with MET and pMET overexpression were eligible for volitinib treatment.

The anti-EGFR monoclonal antibody is approved for colorectal cancer without K-ras mutation, but the EXPAND trial confirmed the limited efficacy of cetuximab in first-line treatment of GC [23]. Further studies indicated that an increased *EGFR* copy number (≥ 4.0) [32] and high EGFR expression on both the mRNA and protein levels [24] were associated with the response of cetuximab. Based on the molecular characteristics of our PDX models, we evaluated the efficacy of two EGFR monoclonal antibodies (BK011 and cetuximab) in five PDX models with different level of EGFR expression or amplification. In our study, both BK011 and cetuximab induced the complete regression of a PDX model with *EGFR* amplification. Studies reported that *EGFR* amplification was found in about 5% of GC [33]; therefore, the EGFR monoclonal antibody should be investigated in further larger GC PDX models with *EGFR* amplification.

Afatinib has been approved as first-line treatment for patients with EGFR mutation-positive NSCLC [34] and second-line treatment for patients with squamous cell carcinoma of the lung [22]. In the phase III LUX-Lung 8 study, the VeriStrat classification (a test measuring acute-phase reactant proteins in the blood) has been reported to be an independent predictor of OS in patients treated with afatinib [35]. Recent research has demonstrated that afatinib monotherapy led to the regression of HER2-amplified GCs by prolonging the inhibition of HER3 and EGFR, which was superior to trastuzumab monotherapy [36]. However, afatinib failed to improve the clinical outcomes in patients with trastuzumab-resistant HER2-overexpressing metastatic breast cancer [37]. The antitumor activity of afatinib in patients with GC has not been explored. In this study, we found that PDX models with *EGFR* amplification, EGFR overexpression, or *HER2* amplification may benefit from afatinib treatment. Afatinib is a pan-HER inhibitor; therefore, further investigation is needed to determine whether afatinib is efficacious in patients with alterations in EGFR family. Patients with *EGFR* amplification, EGFR overexpression or *HER2* amplification should be considered in future clinical trials of afatinib in GC.

Apatinib has been approved as third-line treatment for patients with chemotherapy-refractory AGC. Our preliminary results showed that the efficacy of apatinib was not associated with VEGFR2 expression (data not shown), but the PDX models with high microvessel density were more sensitive to apatinib compared with the PDX models with low CD31 expression. Due to the limited number of available PDX models, the cut-off value of CD31 expression was needed to be defined in the future.

New drugs targeting the cell cycle have become increasingly popular, and the dysregulation of the cell cycle in GC is a frequent event, which suggests the potential therapeutic strategy in clinical practice. It was reported that *CCNE1*, which was amplified in approximately 20% of high-grade serous carcinomas (HGSC) of the ovary, might be a potential biomarker of CDK inhibitors and proteasome inhibitors [38]. In the present study, the CDK1/2/9 inhibitor AZD5438 exerted significant tumor inhibition in two PDX models with high copy number of *CCNE1*, and we also found that AZD5438 exerted antitumor activity by inhibiting the expression of CDK2, CCNE1, and phosphorylated retinoblastoma (pRb). According to our results, although the exact cut-off value of *CCNE1* copy number remains to be validated, patients with high copy numbers of *CCNE1* may benefit from a CDK1/2/9 inhibitor.

As shown in the present study, PDX models retained the high heterogeneity of GC, which is characterized by the activation or suppression of different molecules or pathways. In the future, based on PDX models derived from patients, high-throughput evaluation of multiple potential targeted molecules or pathways will help to screen patients who might be benefit from targeted therapy.

With the exception of the RTK family and its downstream signaling pathway, 18% of the PDX models were EBV-positive, characterized by fewer gene amplifications, a rare mutation of TP53 [33], and the abundant expression of PD-L1 [21], which was consistent with the data from TCGA. A meta-analysis reveals that EBV infection has a favorable impact on the survival of patients with GC, especially in the Asian population [39]. Whether patients with EBV-positive GC may benefit from an immune checkpoint blockade remains uncertain. However, restricted by the absence of an immunodeficient microenvironment, we were not able to evaluate the therapeutic response of immunomodulatory agents in PDX models.

## Conclusions

In conclusion, we have characterized the molecular signatures of our PDX models for GC, which will be useful in future studies, including the exploration of efficacy, predictive markers, combination regimens, and resistant mechanisms.

## Additional files

Additional file 1: **Table S1.** The distribution of Q20 and Q30 representing quality control of sequencing. (DOCX 14 kb)

Additional file 2: **Figure S1.** The work flow of gene variation calling. (DOCX 195 kb)

Additional file 3: Supplementary method. The synthesis details of BK011. (DOCX 13 kb)

Additional file 4: **Figure S2.** In vitro proliferation inhibitory activity of DiFi cells. (DOCX 18 kb)

Additional file 5: **Table S2.** Affinity of BK011 and Erbitux (Cetuximab) for EGFR. (DOCX 13 kb)

Additional file 6: **Table S3.** The list of genes sequenced in this study. (DOCX 16 kb)

Additional file 7: **Table S4.** The clinicalpathological characteristics of xenografts. (DOCX 14 kb)

Additional file 8: **Table S5.** Summary of SNVs, InDels, CNVs, and fusions of the 50 xenografts by targeted sequencing. (DOCX 14 kb)

Additional file 9: **Table S6.** The expression of PD-L1 in EBV-positive and EBV-negative xenografts. (DOCX 12 kb)

## Abbreviations

AGC: Advanced gastric cancer
CNV: Copy number variation
FISH: Fluorescent in situ hybridization
IHC: Immunohistochemistry
InDels: Insertions or deletions
MMR: Mismatch repair
MSI: Microsatellite instable
PDX: Patient-derived xenograft
SNV: Single-nucleotide variant
TCGA: The Cancer Genome Atlas
TGI: Tumor growth inhibition
