# Supplementary material for: Characterization and validation of potential therapeutic targets based on the molecular signature of patient-derived xenografts in gastric cancer
Source: J Hematol Oncol. 2018 Feb 13;11:20. doi: 10.1186/s13045-018-0563-y (PMC5809945; doi:10.1186/s13045-018-0563-y)
Supplement: Supplementary file 1 — Table S1. The distribution of Q20 and Q30 representing quality control of sequencing. (DOCX 14 kb) [file 13045_2018_563_MOESM1_ESM.docx]

**Table S1. The distribution of Q20 and Q30 representing quality control of sequencing.** Q20 and Q30 represented the Quality Control (QC) of sequencing, and the final Q20 and Q30 of all samples were more than 90% and 85%, respectively.

| **PDX ID** | **GC content (%)** | **Q20 (%)** | **Q30 (%)** |
| --- | --- | --- | --- |
| Case004 | 47 | 95.33 | 90.01 |
| Case006 | 47.05 | 95.55 | 90.19 |
| Case007 | 48.14 | 95.09 | 89.59 |
| Case009 | 47.25 | 95.28 | 89.77 |
| Case019 | 46.93 | 95.68 | 90.38 |
| Case025 | 47.23 | 95.75 | 91.06 |
| Case027 | 46.59 | 95.28 | 90.21 |
| Case028 | 46.82 | 95.09 | 89.32 |
| Case037 | 46.74 | 96.77 | 92.71 |
| Case038 | 47.62 | 94.61 | 89.34 |
| Case039 | 46.8 | 96.21 | 91.53 |
| Case042 | 47.31 | 96.64 | 92.64 |
| Case047 | 47.28 | 95.45 | 90.59 |
| Case048 | 46.83 | 95.16 | 89.12 |
| Case050 | 46.72 | 96.15 | 91.42 |
| Case058 | 47.89 | 94.47 | 88.42 |
| Case074 | 46.39 | 95.32 | 89.69 |
| Case075 | 48.8 | 96.62 | 92.45 |
| Case078 | 47.99 | 96.28 | 91.69 |
| Case079 | 47.92 | 95.35 | 90.72 |
| Case082 | 46.6 | 95.97 | 91.14 |
| Case083 | 47.45 | 96.09 | 91.75 |
| Case084 | 45.97 | 94.84 | 88.76 |
| Case086 | 46.4 | 95.45 | 89.97 |
| Case091 | 45.95 | 94.96 | 89 |
| Case099 | 45.89 | 97.19 | 93.73 |
| Case102 | 47.24 | 95.87 | 90.89 |
| Case111 | 47.8 | 95.91 | 90.08 |
| Case117 | 46.36 | 94.98 | 89.06 |
| Case125 | 46.56 | 95.61 | 90.84 |
| Case129 | 46.99 | 96.28 | 91.79 |
| Case131 | 47.05 | 97.03 | 93.19 |
| Case135 | 46.48 | 94.84 | 89.39 |
| Case141 | 47.06 | 95.08 | 89.2 |
| Case142 | 47.32 | 96.62 | 92.52 |
| Case143 | 47.02 | 96.76 | 92.64 |
| Case147 | 47.66 | 94.86 | 88.81 |
| Case148 | 47.76 | 94.56 | 89.35 |
| Case149 | 46.39 | 95 | 89.03 |
| Case152 | 46.38 | 96.03 | 90.41 |
| Case162 | 46.36 | 95.56 | 89.81 |
| Case168 | 46.98 | 95.07 | 89.23 |
| Case174 | 48.05 | 94.47 | 89.06 |
| Case175 | 46.11 | 95.16 | 89.27 |
| Case176 | 47.28 | 95.66 | 90.12 |
| Case191 | 46.33 | 95.34 | 89.59 |
| Case193 | 46.64 | 96.19 | 91.59 |
| Case194 | 47.39 | 95.81 | 91.52 |
| Case203 | 46.48 | 96.05 | 91.16 |
| Case238 | 46.59 | 96.38 | 91.91 |
