# Supplementary figures and images for: Characterization and validation of potential therapeutic targets based on the molecular signature of patient-derived xenografts in gastric cancer
Source: J Hematol Oncol. 2018 Feb 13;11:20. doi: 10.1186/s13045-018-0563-y (PMC5809945; doi:10.1186/s13045-018-0563-y)

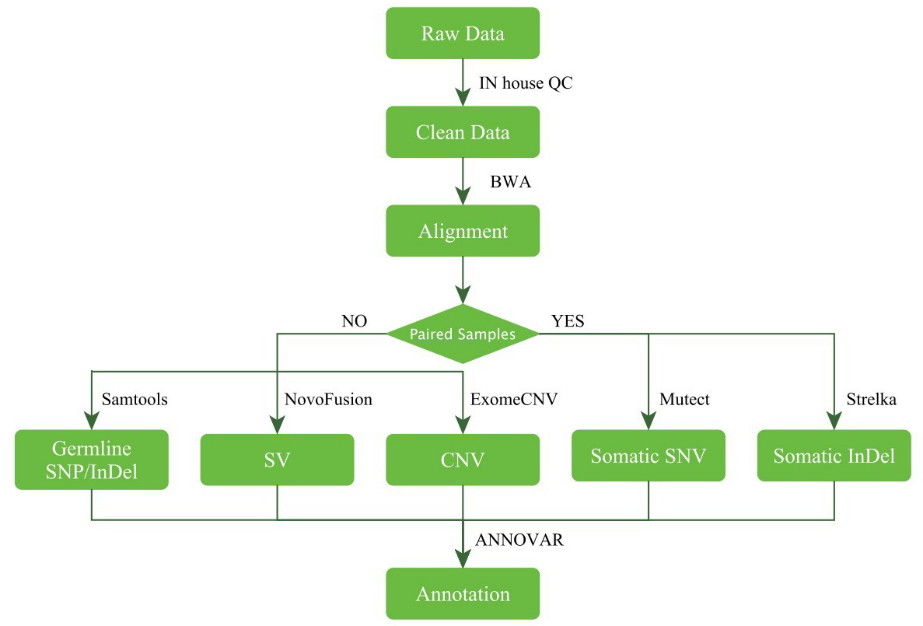

Supplement: Supplementary file 2 — Figure S1. The work flow of gene variation calling. (DOCX 195 kb) [file 13045_2018_563_MOESM2_ESM.docx]
