# Supplementary material for: Characterization and validation of potential therapeutic targets based on the molecular signature of patient-derived xenografts in gastric cancer
Source: J Hematol Oncol. 2018 Feb 13;11:20. doi: 10.1186/s13045-018-0563-y (PMC5809945; doi:10.1186/s13045-018-0563-y)
Supplement: Supplementary file 3 — Supplementary method. The synthesis details of BK011. (DOCX 13 kb) [file 13045_2018_563_MOESM3_ESM.docx]

**Supplementary Methods**

**Construction of engineering cell lines**

Gene sequences with signal peptide coding sequences were designed according to the amino acid sequence of heavy chain and light chain of Erbitux (Cetuximab) and were cloned into expression vector PBK01, which had two expression units. The recombinant expression plasmid pBK01-BK011 were then co-transfected with plasmid pBK03, which was labeled with selectable marker dhfr, into CHO DG44 cells. The transfected cells were cultured in 96-well plates and clones with high expression of BK011, which as detected by ELISA in the supernatant, were selected and were then subjected to MTX pressure to further increase the yield. These clones were then subcloned by limiting dilution to obtain BK011 engineered cell lines.

**Establishment of production process**

The serum-free flow medium for BK-11 engineered cell line was selected by basal medium screening followed by flow medium screening. Specifically, 1 x10^6^ cells were seeded in JS CD012 medium with pH set point of 6.9 ± 0.1. Then, 5%, 2%, 5% and 2% JS CD feed was added on day 4, 6, 8 and 10. Glucose was measured daily from day 3 and was added to 6 g/L when less than 2 g/L. The total incubation time was 14 days. The purification of BK011 was established by comprehensive investigation of protein yield and purity. Clarification => Mabselect SuRe affinity chromatography => Low pH virus inactivation => Capto S cation exchange => Capto Q Anion exchange => nanofiltration => Sterilization filter => stock solution.
