# Supplementary material for: Characterization and validation of potential therapeutic targets based on the molecular signature of patient-derived xenografts in gastric cancer
Source: J Hematol Oncol. 2018 Feb 13;11:20. doi: 10.1186/s13045-018-0563-y (PMC5809945; doi:10.1186/s13045-018-0563-y)
Supplement: Supplementary file 5 — Table S2. Affinity of BK011 and Erbitux (Cetuximab) for EGFR. (DOCX 13 kb) [file 13045_2018_563_MOESM5_ESM.docx]

**Table S2. Affinity of BK011 and Erbitux (Cetuximab) for EGFR**

|  | ka(1/Ms) | kd(1/s) | KA(1/M) | KD(M) |
| --- | --- | --- | --- | --- |
| Erbitux | 8.83E+05 | 3.93E-04 | 2.25E+09 | 4.45E-10 |
| BK011 | 2.40E+06 | 2.35E-04 | 1.02E+10 | 9.78E-11 |
