# Supplementary material for: Characterization and validation of potential therapeutic targets based on the molecular signature of patient-derived xenografts in gastric cancer
Source: J Hematol Oncol. 2018 Feb 13;11:20. doi: 10.1186/s13045-018-0563-y (PMC5809945; doi:10.1186/s13045-018-0563-y)
Supplement: Supplementary file 7 — Table S4. The clinicalpathological characteristics of xenografts. (DOCX 14 kb) [file 13045_2018_563_MOESM7_ESM.docx]

**Table S4.** **The clinicalpathological characteristics of xenografts.** The differentiation level, Lauren classification, chemotherapy status and HER2 expression of the 50 xenografts.

| **PDX ID** | **Differentiation** | **Lauren Classification** | **Chemotherapy**  **Status** | **HER2 Expression** |
| --- | --- | --- | --- | --- |
| Case004 | Good | Intestinal | Post | Negative |
| Case006 | Good | Intestinal | Post | Positive |
| Case007 | Good | Intestinal | Pre | Negative |
| Case009 | Good | Intestinal | Pre | Negative |
| Case019 | Good | Intestinal | Post | Positive |
| Case025 | Poor | Diffuse | Post | Negative |
| Case027 | Poor | Diffuse | Pre | Negative |
| Case028 | Good | Intestinal | Post | Negative |
| Case037 | Good | Intestinal | Pre | Negative |
| Case038 | Good | Intestinal | Post | Negative |
| Case039 | Poor | Intestinal | Pre | Negative |
| Case042 | Poor | Mixed | Pre | Negative |
| Case047 | Poor | Intestinal | Pre | Negative |
| Case048 | Poor | Diffuse | Pre | Negative |
| Case050 | Poor | Diffuse | Pre | Negative |
| Case058 | Poor | Diffuse | Pre | Negative |
| Case074 | Poor | Diffuse | Pre | Negative |
| Case075 | Poor | Diffuse | Pre | Negative |
| Case078 | Good | Intestinal | Pre | Negative |
| Case079 | Poor | Diffuse | Post | Negative |
| Case082 | Good | Intestinal | Pre | Negative |
| Case083 | Poor | Diffuse | Pre | Negative |
| Case084 | Good | Intestinal | Post | Negative |
| Case086 | Poor | Intestinal | Pre | Negative |
| Case091 | Poor | Diffuse | Post | Negative |
| Case099 | Poor | Diffuse | Post | Negative |
| Case102 | Poor | Diffuse | Post | Negative |
| Case111 | Poor | Diffuse | Pre | Negative |
| Case117 | Poor | Diffuse | Post | Negative |
| Case125 | Poor | Intestinal | Pre | Negative |
| Case129 | Poor | Diffuse | Post | Negative |
| Case131 | Poor | Diffuse | Post | Negative |
| Case135 | Poor | Diffuse | Post | Negative |
| Case141 | Poor | Diffuse | Post | Negative |
| Case142 | Good | Intestinal | Post | Positive |
| Case143 | Poor | Intestinal | Post | Negative |
| Case147 | Good | Intestinal | Pre | Negative |
| Case148 | Good | Intestinal | Pre | Negative |
| Case149 | Poor | Diffuse | Pre | Negative |
| Case152 | Poor | Diffuse | Pre | Negative |
| Case162 | Poor | Diffuse | Pre | Negative |
| Case168 | Good | Intestinal | Pre | Negative |
| Case174 | Poor | Diffuse | Post | Negative |
| Case175 | Poor | Intestinal | Post | Negative |
| Case176 | Good | Intestinal | Post | Positive |
| Case191 | Poor | Mixed | Pre | Negative |
| Case193 | Poor | Diffuse | Post | Negative |
| Case194 | Good | Intestinal | Post | Negative |
| Case203 | Good | Intestinal | Post | Negative |
| Case238 | Poor | Intestinal | Post | Negative |
