# Supplementary material for: Characterization and validation of potential therapeutic targets based on the molecular signature of patient-derived xenografts in gastric cancer
Source: J Hematol Oncol. 2018 Feb 13;11:20. doi: 10.1186/s13045-018-0563-y (PMC5809945; doi:10.1186/s13045-018-0563-y)
Supplement: Supplementary file 8 — Table S5. Summary of SNVs, InDels, CNVs, and fusions of the 50 xenografts by targeted sequencing. (DOCX 14 kb) [file 13045_2018_563_MOESM8_ESM.docx]

**Table S5. Summary of SNVs, InDels, CNVs, and Fusions of the 50 xenografts by targeted sequencing.** In total, 1325 variations in xenografts from human sequence reads were called, including 581 nonsynonymous single nucleotide variations (SNVs), 225 amplifications, 513 indels and 6 translocation fusions.

| **ID** | **SNV** | **InDels** | **CNV** | **Fusion** |
| --- | --- | --- | --- | --- |
| Case004 | 11 | 11 | 9 | 0 |
| Case006 | 20 | 19 | 7 | 0 |
| Case007 | 5 | 17 | 4 | 0 |
| Case009 | 14 | 3 | 5 | 0 |
| Case019 | 6 | 3 | 2 | 2 |
| Case025 | 18 | 6 | 11 | 0 |
| Case027 | 12 | 5 | 0 | 0 |
| Case028 | 13 | 16 | 8 | 0 |
| Case037 | 10 | 7 | 1 | 0 |
| Case038 | 11 | 20 | 0 | 0 |
| Case039 | 7 | 4 | 4 | 0 |
| Case042 | 11 | 17 | 9 | 2 |
| Case047 | 13 | 10 | 3 | 0 |
| Case048 | 15 | 17 | 7 | 0 |
| Case050 | 6 | 9 | 0 | 0 |
| Case058 | 1 | 6 | 0 | 0 |
| Case074 | 6 | 12 | 0 | 0 |
| Case075 | 12 | 5 | 8 | 0 |
| Case078 | 19 | 7 | 11 | 0 |
| Case079 | 10 | 23 | 8 | 0 |
| Case082 | 36 | 31 | 2 | 0 |
| Case083 | 43 | 11 | 1 | 0 |
| Case084 | 11 | 15 | 1 | 0 |
| Case086 | 10 | 2 | 16 | 0 |
| Case091 | 14 | 8 | 0 | 0 |
| Case099 | 16 | 10 | 19 | 0 |
| Case102 | 9 | 12 | 9 | 2 |
| Case111 | 8 | 6 | 13 | 0 |
| Case117 | 7 | 7 | 1 | 0 |
| Case125 | 10 | 12 | 8 | 0 |
| Case129 | 9 | 7 | 1 | 0 |
| Case131 | 9 | 4 | 2 | 0 |
| Case135 | 7 | 9 | 0 | 0 |
| Case141 | 11 | 4 | 8 | 0 |
| Case142 | 9 | 17 | 8 | 0 |
| Case143 | 15 | 13 | 3 | 0 |
| Case147 | 5 | 11 | 10 | 0 |
| Case148 | 3 | 6 | 0 | 0 |
| Case149 | 18 | 7 | 2 | 0 |
| Case152 | 12 | 14 | 0 | 0 |
| Case162 | 10 | 8 | 0 | 0 |
| Case168 | 9 | 9 | 3 | 0 |
| Case174 | 3 | 2 | 0 | 0 |
| Case175 | 9 | 6 | 7 | 0 |
| Case176 | 18 | 10 | 4 | 0 |
| Case191 | 6 | 10 | 0 | 0 |
| Case193 | 5 | 13 | 3 | 0 |
| Case194 | 9 | 19 | 1 | 0 |
| Case203 | 13 | 7 | 1 | 0 |
| Case238 | 17 | 6 | 5 | 0 |
