# Supplementary material for: Characterization and validation of potential therapeutic targets based on the molecular signature of patient-derived xenografts in gastric cancer
Source: J Hematol Oncol. 2018 Feb 13;11:20. doi: 10.1186/s13045-018-0563-y (PMC5809945; doi:10.1186/s13045-018-0563-y)
Supplement: Supplementary file 9 — Table S6. The expression of PD-L1 in EBV-positive and EBV-negative xenografts. (DOCX 12 kb) [file 13045_2018_563_MOESM9_ESM.docx]

**Table S6. The expression of PD-L1 in EBV-positive and EBV-negative xenografts.** The positive rate of PD-L1 expression was higher in EBV-positive xenografts than EBV-negative xenografts.

| PD-L1 expression | EBV infection | | P value |
| --- | --- | --- | --- |
|  | Positive | Negative |  |
| Positive | 7 | 9 | 0.004 |
| Negative | 2 | 32 |  |
