# Supplementary material for: Characterization and validation of potential therapeutic targets based on the molecular signature of patient-derived xenografts in gastric cancer
Source: J Hematol Oncol. 2018 Feb 13;11:20. doi: 10.1186/s13045-018-0563-y (PMC5809945; doi:10.1186/s13045-018-0563-y)
Supplement: Supplementary file 9 [file 13045_2018_563_MOESM4_ESM.docx]

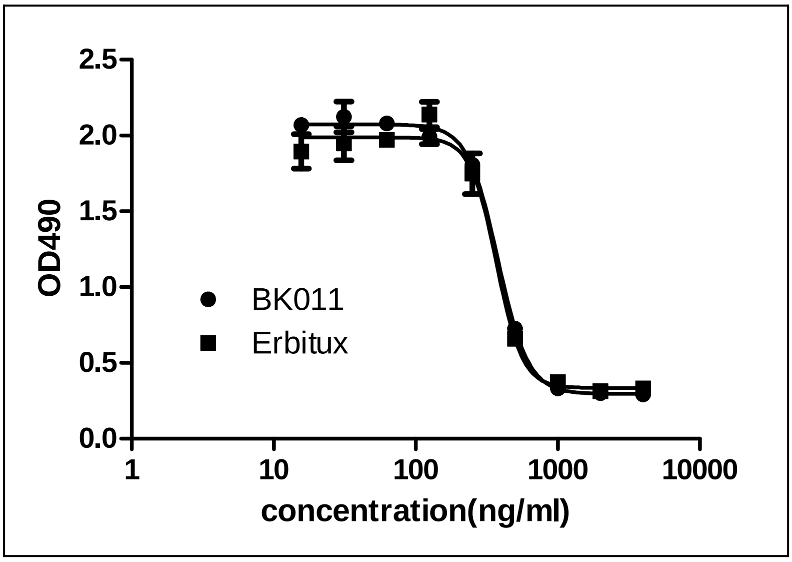

2.5
2.0
1.5
1.0
0.5
0.0
OD490
BK011
Erbitux
1
10
100
1000
10000
concentration(ng/ml)
